# Effect of different treatment modalities on the prognosis of stage IV epithelial ovarian cancer: analysis of the SEER database

**DOI:** 10.1186/s12905-024-03199-5

**Published:** 2024-06-15

**Authors:** Shuyuan Zhang, Hongyong Zhang, Naer Jia, Suo Suo, Jianfeng Guo

**Affiliations:** 1 Department of Obstetrics and Gynecology, Union Hospital, Tongji Medical College, Huazhong University of Science and Technology, No 1277 JieFang Avenue, Jiang’an District, Wuhan, 420022 China; 2 Department of Hematology, Union Hospital, Tongji Medical College, Huazhong University of Science and Technology, Wuhan, China; 3 Department of Obstetrics and Gynecology, People’s Hospital Of Bortala Mongolian Autonomous Prefecture, Bortala Mongolian Autonomous Prefecture, Xinjiang Uygur Autonomous Region, China; 4 People’s Hospital of Longhua, Shenzhen, China; 5 Longhua District Key Laboratory of Perinatal Population Medicine, Shenzhen, China

**Keywords:** Carcinoma, Ovarian epithelial, Surgery, Radiotherapy, Chemotherapy, SEER database

## Abstract

**Background:**

The prognosis of advanced ovarian cancer is often poor. Although there are several treatment options for stage IV epithelial ovarian cancer, it is not clear which treatment will benefit the patient’s prognosis.We conducted an analysis using the SEER database to compare the impact of different treatment modalities on the prognosis of advanced ovarian cancer.

**Methods:**

The present study conducts a retrospective analysis of relevant data from the SEER database pertaining to patients diagnosed with stage IV epithelial ovarian cancer between 2011 and 2020 (*n* = 5345). Statistical methods including Kaplan-Meier curves, log-rank tests, and Cox regression analysis are employed to ascertain the impact of different treatment regimens on the prognosis of patients with stage IV epithelial ovarian cancer.

**Results:**

Among patients with stage IV epithelial ovarian cancer, age ≥ 60 and the presence of lung metastases or multiple metastases were identified as poor prognostic factors. Conversely, being Asian or Pacific Islander, married, and testing negative for CA125 were associated with favorable prognoses. In terms of the choice of treatment for patients, surgery plus chemotherapy was the best treatment modality, and timely surgery could significantly improve the prognosis of patients, but there was no difference between chemoradiotherapy alone and the surgery group among patients with lung metastases.

**Conclusion:**

The prognosis of patients with stage IV epithelial ovarian cancer is influenced by many factors. In terms of the choice of treatment, patients with surgery plus chemotherapy have the best prognosis. In cases where lung metastases are inoperable, a combination of radiotherapy and chemotherapy can be used. In other cases, radiotherapy does not improve outcomes in patients with stage IV epithelial ovarian cancer. This study provides a basis for the choice of treatment for patients with stage IV epithelial ovarian cancer.

## Introduction

Ovarian cancer is the fourth leading cause of cancer-related deaths in women. In 2024, according to statistics from the United States, there were 19,680 new cases of ovarian cancer and 12,740 deaths [1]. The World Health Organization classifies the histological types of ovarian cancers resulting in probability of origin: epithelial, germ cell, gonadal mesenchymal, metastatic, and other types [2]. Epithelial ovarian cancer is the most common histological type of ovarian cancer, accounting for 65% of cases. Due to the absence of typical early symptoms and the lack of effective screening methods, most patients have already developed retroperitoneal or distant metastases at the time of initial diagnosis [3]. Stage IV epithelial ovarian cancer is a complex and challenging disease with multiple economic, social, and behavioral dimensions. First of all, the medical costs required to treat the disease are huge, including the cost of surgery, chemotherapy, radiation and other treatments, which puts a heavy burden on patients and medical insurance. Second, due to late diagnosis or delayed treatment, patients with stage IV epithelial ovarian cancer often require prolonged hospitalization, resulting in loss of work capacity and productivity, which in turn affects patients’ socioeconomic status. In addition, the clinical symptoms of the disease may trigger psychological burden and anxiety in patients, negatively affecting their quality of life.

Currently, the prognosis of patients with epithelial ovarian cancer with distant metastases is poor, and there are several options for the treatment of these patients. The selection of treatment regimens necessitates a comprehensive consideration of factors such as disease stage, patient age, physical condition, life expectancy, and comorbidities. Current treatment modalities include surgery, chemotherapy, radiotherapy, novel targeted molecular therapies, or their combinations. Surgery plus postoperative adjuvant chemotherapy is the main treatment option [4]. Despite advancements in ovarian cancer treatment, such as extensive cytoreductive surgery and novel adjunctive therapies, the overall survival rate for stage III patients remains relatively low at 40%, with even lower rates for stage IV patients at 20%. However, with advancements in modern surgery and chemotherapy for ovarian cancer, the median survival and overall survival rates for late-stage patients have improved over the past 15 years [5].

Some studies suggest that radiotherapy may be a potential treatment option for advanced ovarian cancer, and modern radiotherapy may still play a role in certain pathological types of ovarian cancer [6, 7]. Epithelial ovarian cancer, particularly clear cell carcinoma, is a radiosensitive cancer that can benefit from radiotherapy: clear cell carcinoma is usually confined to the pelvis and is resistant to chemotherapy, thus radiotherapy can be advantageous in terms of local control of the lesion and reducing the recurrence of ovarian cancer [6, 7]. However, the role of radiotherapy in the management of ovarian cancer remains a controversial topic, and the role of radiotherapy in the treatment of patients with stage IV ovarian cancer with lung metastases may be of interest. The main treatment modality for patients with epithelial ovarian cancer in the presence of lung metastases is surgery or radiotherapy alone is still unclear. Therefore, the role of radiotherapy in the adjuvant treatment of ovarian cancer patients, particularly those with epithelial ovarian cancer, deserves further study. Therefore, the choice of treatment modality for patients with advanced epithelial ovarian cancer is particularly important.

After the diagnosis of ovarian cancer, a combination of appropriate treatment options is required based on the stage of the disease, age, physical performance status, life expectancy and comorbidities. Although there are many treatment options for patients with advanced ovarian cancer, there is still a lot of uncertainty about how to choose the treatment that will benefit the patient’s prognosis. Therefore, there is a need to find the best treatment for patients with advanced ovarian cancer. In this paper, we analyzed the different treatment options for patients with stage IV epithelial ovarian cancer (staging criteria: Derived AJCC Stage Group, 7th ed (2011–2015), 7th Edition Stage Group Recode (2016–2017), Derived EOD 2018 Stage Group (2018–2020)), to explore the impact of the different treatment options on the prognosis of patients with stage IV epithelial ovarian cancer. We collected data related to 5345 patients with stage IV epithelial ovarian cancer from SEER database between 2011 and 2020 and investigated the impact of the choice of different treatment options on the prognosis of patients with stage IV epithelial ovarian cancer through statistical analysis, which will offer novel insights into clinical decision-making regarding treatment options for patients with stage IV epithelial ovarian cancer in the future.

## Methods

### Data source

The data in this article originates from the SEER (Surveillance, Epidemiology, and End Results) database, which is a public database and research resource created by the National Cancer Institute (NCI). The SEER database collects and stores cancer incidence, survival, and treatment data from the United States to support cancer research and epidemiological investigations. The SEER database collects and stores cancer incidence, survival, and treatment data from across the United States with the aim of supporting cancer research and epidemiological investigations. We obtained permission to access the SEER database and extracted data of 5,345 patients from the SEER*Stat software (version 8.3.8).

### Study population

**Fig. 1:**
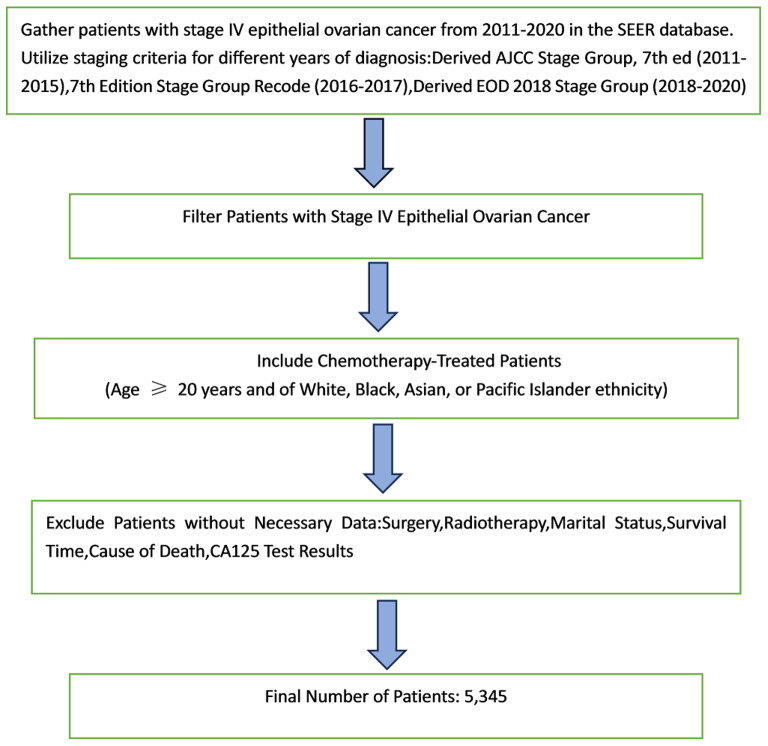
Experimental flow chart

We collected patients with stage IV epithelial ovarian cancer from 2011 to 2020 in the SEER database based on staging criteria for different years of diagnosis (Derived AJCC Stage Group, 7th ed (2011–2015), 7th Edition Stage Group Recode (2016–2017), Derived EOD 2018 Stage Group (2018–2020)) were collected from 2011 to 2020 for patients with stage IV epithelial ovarian cancer, and chemotherapy-treated patients who were aged ≥ 20 year and of White, Black, Asian, or Pacific Islander ethnicity were included in the statistics, and those without surgery/radiotherapy/marital status/survival time/cause of death/ CA125 test results were excluded, resulting in a final number of 5,345 patients included in the statistics (Figure 1).

### Survival

Survival times for this study were determined based on the cancer-specific survival of the patients. Cancer-specific survival refers to the time from the date of diagnosis to the date of ovarian cancer-related death. Patients who died or were lost to follow-up for other reasons were considered censored data.

### Statistical analysis

This study collected data on diagnosis year, age, race, marital status, CA125, sites of metastasis, and survival time.All analyses were conducted within the SEER database. Survival distributions were grouped by treatment modality in the overall study population and compared using Kaplan-Meier curves and log-rank tests. Hazard ratios (HRs) and 95% confidence intervals (CIs) for multivariable adjusted associations between treatment modality and cancer-specific mortality in the overall study population were estimated using Cox proportional risk models. Adjustment factors included year of diagnosis, age, race, marital status, and CA125.Statistical analyses in this study were conducted in GraphPad Prism versions 8.0.2 and R 4.2.1.

## Results

This study included 5345 patients with stage IV epithelial ovarian cancer. Table 1 depicts the overall characteristics of patients with stage IV epithelial ovarian cancer and the total number of cancer-specific deaths under different treatment modalities (classified as surgery plus chemotherapy, surgery plus radiotherapy, chemotherapy alone, and radiotherapy alone), according to the database (Table 1).

**Table 1:** Characteristics of women with stage IV ovarian cancer overall and by treatment type according to database

	Surgery plus chemotherapy	Surgery plus chemoradiotherapy	Only chemotherapy	Only chemoradiotherapy	Totality
	Overall (Deaths)	Overall (Deaths)	Overall (Deaths)	Overall (Deaths)	Overall (Deaths)
**Year of diagnosis**					
2011–2015	1995(1440)	22(17)	453(386)	14(11)	2484(1854)
2016–2020	2234(699)	35(11)	573(352)	19(10)	2861(1072)
**Age(>20)**					
≤ 59	1597(787)	29(15)	223(156)	13(11)	1862(969)
≥ 60	2632(1352)	28(13)	803(582)	20(10)	3483(1957)
**Race**					
Black	322(174)	9(5)	141(103)	2(1)	474(283)
White	3490(1773)	39(18)	819(594)	27(18)	4375(2403)
Asian or Pacific Islander	417(192)	9(5)	66(41)	4(2)	496(240)
**Marital status at diagnosis**					
Married (including common law)	2375(1167)	26(12)	450(319)	17(12)	2868(1510)
others	1854(972)	31(16)	576(419)	16(9)	2477(1416)
**CA-125**					
Postive	4124(2102)	53(26)	1004(726)	29(19)	5210(2873)
Negative	105(37)	4(2)	22(12)	4(2)	135(53)
**Site of metastasis**					
No metastases in all 4 organs	2732(1326)	27(10)	564(386)	6(6)	3329(1728)
Mets at DX-bone	35(13)	4(3)	14(9)	5(3)	58(28)
Mets at DX-brain	1(0)	2(0)	5(4)	4(2)	12(6)
Mets at DX-liver	711(347)	7(5)	197(141)	2(1)	917(494)
Mets at DX-lung	483(287)	9(4)	114(88)	5(2)	611(381)
Multiple metastases (≥ 2 metastatic sites)	267(166)	8(6)	132(110)	11(7)	418(289)
**Survival months**					
0–50	3337(1825)	51(26)	958(707)	31(20)	4377(2578)
51–100	800(305)	5(2)	61(31)	2(1)	868(339)
101–150	92(9)	1(0)	7(0)	0(0)	100(9)
**Totality**	4229(2139)	57(28)	1026(738)	33(21)	5345(2926)

**Fig. 2:**
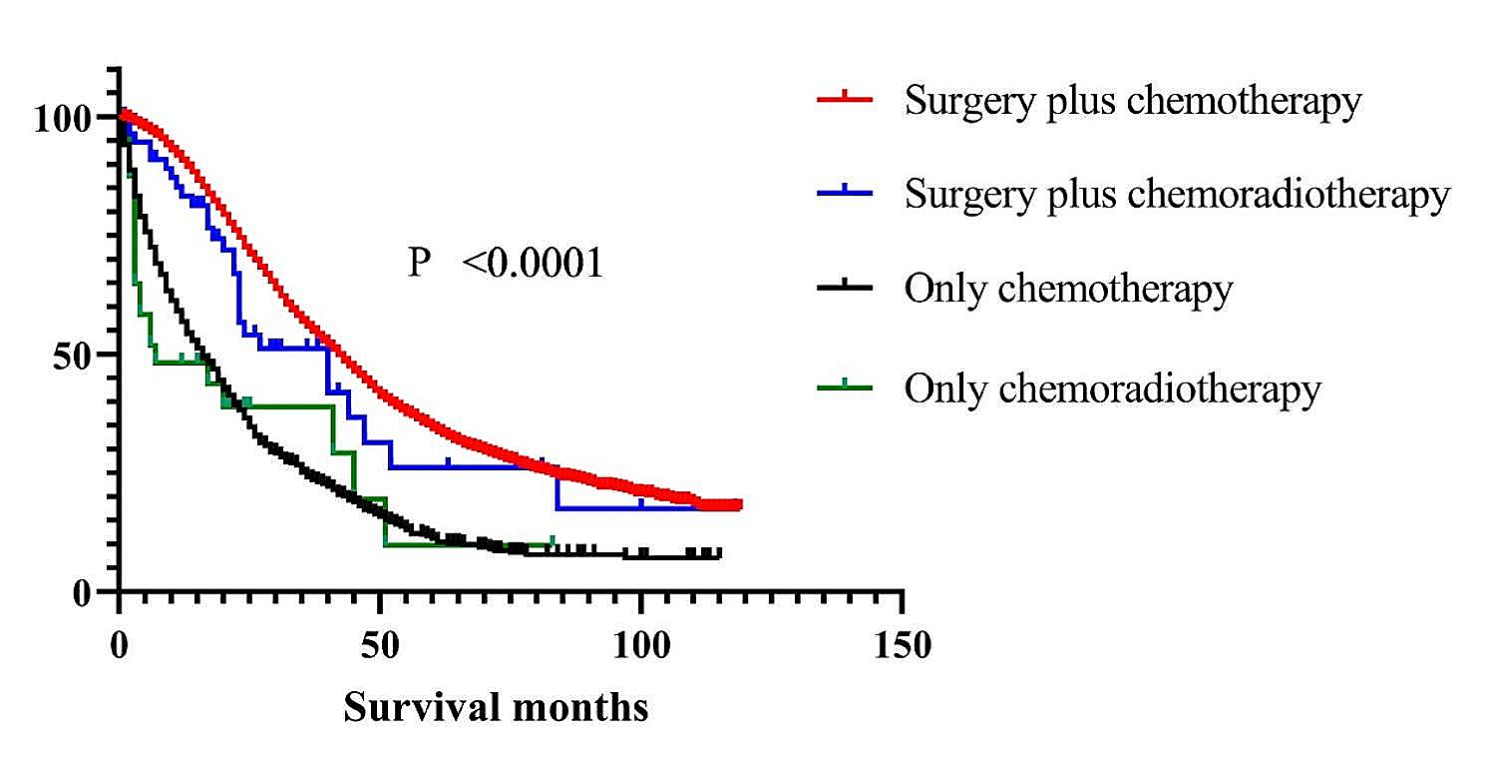
Kaplan-Meier curves for different treatment modalities and their corresponding survival times. Treatment modality was significantly associated with survival time in all groups (*P* < 0.0001)

By comparing the overall survival rate of patients with stage IV ovarian cancer with different treatments, We found that patients in the surgical group had a longer survival time than those in the non-surgical group (Figure 2). In the surgical group, surgery plus chemotherapy was the most effective in improving the prognosis of patients with stage IV epithelial ovarian cancer.

We then analysed the multivariate corrected hazard ratios (HRs) and 95% confidence intervals (CIs) for stage IV epithelial ovarian cancer with different prognostic factors. When discussing the different treatment modalities, we used surgery plus chemotherapy as the control group, then the adjusted HR for surgery plus radiotherapy was 1.326 (0.910–1.932), *P* = 0.142, indicating it was not a risk factor. The adjusted HR for chemotherapy alone was 2.786 (2.553–3.041), *P* < 0.001; and the adjusted HR for radiotherapy alone was 3.003 (1.944–4.638), *P* < 0.001, so timely surgery can improve the prognosis of patients with stage IV epithelial ovarian cancer. When discussing different ages, with age ≤ 59 as the control group, the adjusted HR for age ≥ 60 was 1.214 (1.122–1.313), *P* < 0.001.When discussing different metastatic sites, with no metastases in all 4 organs as the control group, the adjusted HR for lung metastases was 1.151 (1.030–1.287), *P* = 0.013, and the adjusted HR for multiple metastases (≥ 2 metastatic sites) was 1.417 (1.250–1.606), *P* < 0.001. When discussing different races, using Black as the control group, the adjusted HR for Asian or Pacific Islander was 0.821 (0.689–0.977), *p* = 0.026.When discussing marital status, using married (including common law) as the control group, the adjusted HR for others was 1.154 (1.071–1.242), *p* < 0.001.When discussing CA125, with CA125 positive as the control group, the adjusted HR for CA125 negative was 0.611 (0.464–0.804), *p* < 0.001.Meanwhile age ≥ 60, presence of lung metastasis or multiple metastases were identified as poor prognostic factors for stage IV epithelial ovarian cancer (*P* < 0.05). While being Asian or Pacific Islander, married, and CA125 negative were favourable prognostic factors for patients with stage IV epithelial ovarian cancer (*P* < 0.05) (Table 2).

**Table 2:** Univariable and multivariable-adjusted hazard ratios (HRs) and 95% confidence intervals for the association between all factors and overall survival in the overall study population

	Death/Overall(%)	HR(95% CI)	*P* value	HR(95% CI) Multivariate analysis	*P* value Multivariate analysis
**Treatment method**					
Surgery plus chemotherapy	2139/4229(50.58%)	Reference		Reference	
Surgery plus chemoradiotherapy	28/57(49.12%)	1.289 (0.888–1.872)	0.182	1.326 (0.910–1.932)	0.142
Only chemotherapy	738/1026(71.93%)	2.910 (2.674–3.167)	< 0.001	2.786 (2.553–3.041)	< 0.001
Only chemoradiotherapy	21/33(63.64%)	3.168 (2.061–4.870)	< 0.001	3.003 (1.944–4.638)	< 0.001
**Year of diagnosis**					
2011–2015	1854/2484(74.64%)	Reference			
2016–2020	1072/2861(37.47%)	1.038 (0.958–1.124)	0.364		
**Age(≥20)**					
≤ 59	969/1862(52.04%)	Reference		Reference	
≥ 60	1957/3483(56.18%)	1.337 (1.238–1.444)	< 0.001	1.214 (1.122–1.313)	< 0.001
**Race**					
Black	283/474(59.70%)	Reference		Reference	
White	2403/4375(54.92%)	0.818 (0.723–0.925)	0.001	0.909 (0.802–1.031)	0.136
Asian or Pacific Islander	240/496(48.39%)	0.702 (0.591–0.833)	< 0.001	0.821 (0.689–0.977)	0.026
**Marital status at diagnosis**					
Married (including common law)	1510/2868(52.65%)	Reference		Reference	
others	1416/2477(57.16%)	1.262 (1.173–1.357)	< 0.001	1.154 (1.071–1.242)	< 0.001
**CA-125**					
Positive	2873/5210(55.14%)	Reference		Reference	
Negative	53/135(39.25%)	0.623 (0.475–0.818)	< 0.001	0.611 (0.464–0.804)	< 0.001
**Site of metastasis**					
No metastases in all 4 organs	1728/3329(51.91%)	Reference		Reference	
Mets at DX-bone	28/58(48.28%)	0.975 (0.671–1.417)	0.896	0.859 (0.590–1.253)	0.431
Mets at DX-brain	6/12(50.00%)	1.482 (0.665–3.303)	0.337	0.909 (0.403–2.049)	0.818
Mets at DX-liver	494/917(53.87%)	0.998 (0.903–1.103)	0.969	0.997 (0.902–1.102)	0.959
Mets at DX-lung	381/611(62.36%)	1.164 (1.041–1.300)	0.007	1.151 (1.030–1.287)	0.013
Multiple metastases (≥ 2 metastatic sites)	289/418(69.14%)	1.537 (1.357–1.741)	< 0.001	1.417 (1.250–1.606)	< 0.001

We further investigated the impact of four treatment modalities on the prognosis of patients with stage IV epithelial ovarian cancer in the presence of adverse pathological prognostic factors (age ≥ 60 years, lung metastases and multiple sites, and CA125 positivity). We found that patients who underwent surgery had a better prognosis compared to non-surgical patients. However, among patients with lung metastases, there was no difference between chemoradiotherapy alone and surgery group, with an adjusted HR of 0.845 (0.207–3.454), *p* = 0.814 (Table 3).

**Table 3:** Univariable and multivariable-adjusted hazard ratios (HRs) and 95% confidence intervals for the association between treatment and overall survival stratified by poor-prognostic factors, according to database

	**Age ≥ 60(** ***n*** ** = 3483)**				
Treatment method	Death/Overall(%)	HR(95% CI)	*P* value	HR(95% CI) Multivariate analysis	*P* value Multivariate analysis
Surgery plus chemotherapy	1352/2632(51.37%)	Reference		Reference	
Surgery plus chemoradiotherapy	13/28(46.43%)	1.062 (0.615–1.834)	0.829	1.113 (0.642–1.930)	0.703
Only chemotherapy	582/803(72.48%)	2.791 (2.530–3.079)	< 0.001	2.710 (2.454–2.994)	< 0.001
Only chemoradiotherapy	10/20(50.00%)	2.152 (1.155–4.011)	0.016	2.076 (1.110–3.884)	0.022
	**Mets at DX-lung(** ***n*** ** = 611)**				
Treatment method	Death/Overall(%)	HR(95% CI)	*P* value	HR(95% CI) Multivariate analysis	*P* value Multivariate analysis
Surgery plus chemotherapy	287/483(59.42%)	Reference		Reference	
Surgery plus chemoradiotherapy	4/9(44.44%)	0.859 (0.320–2.304)	0.762	0.945 (0.351–2.545)	0.911
Only chemotherapy	88/114(77.19%)	2.701 (2.123–3.437)	< 0.001	2.517 (1.955–3.242)	< 0.001
Only chemoradiotherapy	2/5(40.00%)	0.855 (0.213–3.441)	0.826	0.845 (0.207–3.454)	0.814
	**Multiple metastases(** ***n*** ** = 418)**				
Treatment method	Death/Overall(%)	HR(95% CI)	*P* value	HR(95% CI) Multivariate analysis	*P* value Multivariate analysis
Surgery plus chemotherapy	166/267(62.17%)	Reference		Reference	
Surgery plus chemoradiotherapy	6/8(75.00%)	2.758 (1.213–6.272)	0.015	2.530 (1.107–5.781)	0.028
Only chemotherapy	110/132(83.33%)	3.400 (2.642–4.377)	< 0.001	3.233 (2.503–4.177)	< 0.001
Only chemoradiotherapy	7/11(63.64%)	3.822 (1.774–8.236)	< 0.001	3.670 (1.700–7.926)	< 0.001
	**CA-125 positive(** ***n*** ** = 5210)**				
Treatment method	Death/Overall(%)	HR(95% CI)	*P* value	HR(95% CI) Multivariate analysis	*P* value Multivariate analysis
Surgery plus chemotherapy	2102/4124(50.97%)	Reference		Reference	
Surgery plus chemoradiotherapy	26/53(49.06%)	1.305 (0.887–1.922)	0.177	1.281 (0.864–1.899)	0.219
Only chemotherapy	726/1004(72.31%)	2.954 (2.712–3.217)	< 0.001	2.794 (2.556–3.053)	< 0.001
Only chemoradiotherapy	19/29(65.52%)	3.008 (1.914–4.726)	< 0.001	2.773 (1.754–4.385)	< 0.001

## Discussion

Our current study confirms that patients with stage IV epithelial ovarian cancer treated with surgery plus chemotherapy exhibit the most favorable prognosis, with a median survival time of 41 months. While the National Comprehensive Cancer Network(NCCN)guidelines recommend tumor cytoreduction for stage IV epithelial ovarian cancer patients [8], a recent econometric analysis of 46 studies, involving 18,579 patients, evaluating predictors of 30-day mortality in those undergoing tumor cytoreduction for ovarian cancer, demonstrated that the combined effects of increasing age and advanced clinical staging factors significantly increase the risk of perioperative mortality [9]. However, various studies have shown a survival benefit of complete cytoreduction irrespective of age [10]. Moreover, studies have indicated that patients with epithelial ovarian cancer involving the liver, biliary tract, or hilum, undergoing complete cytoreduction experience a survival benefit [11]. Our study further confirms the above studies.

The role of lymph node dissection following tumor cytoreduction in ovarian cancer remains a topic of controversy. The current study suggests that lymph node dissection in early-stage EOC does not confer a survival benefit for patients. A multicenter randomized trial evaluating the value of systematic lymph node dissection in early-stage EOC revealed no statistically significant difference in 5-year overall survival between the lymph node dissection and control groups (5-year OS 84.0% vs. 81.6%) [12]. However, patients with advanced ovarian cancer exhibit a higher incidence of pelvic and para-aortic lymph node metastases [13]. Thus, studies have demonstrated that patients with advanced ovarian cancer who undergo lymph node dissection experience a significant survival advantage [14, 15].

Consistent with these studies, in our study, elderly patients over 60 years of age still benefited from surgery. However, it has been suggested that older patients, particularly those aged over 80 years, are less likely to undergo surgery and achieve optimal tumour reduction [16]. In older patients, aggressive surgery may lead to shorter survival, especially in those with poorer general health conditions. There is also concern that adverse effects after surgery may hinder older patients from receiving chemotherapy. A retrospective report showed that among 85 patients aged over 80 years who underwent tumour cytoreduction, of whom 13% died before discharge and 20% within 60 days of surgery. Furthermore, 13% never received adjuvant therapy, and 43% of these patients completed less than 3 cycles of therapy [17]. Therefore, the choice of treatment for elderly patients with advanced ovarian cancer cannot be generalized, but should be based on a physical status assessment, a “Geriatric Vulnerability Score (GVS)” developed by the French National Group of Investigators for the Study of Ovarian and Breast Cancer (GINECO). In this score, FIGO stage IV, physical status ≥ 2, age > 80 years, Activities of Daily Living(ADL)score < 6,Instrumental Activity of Daily Living (IADL) score < 25, 3 or more comorbidities, albumin < 35 g/L, and lymphocytes < 1G/L are statistically associated with poor survival [18]. Individualized therapeutic regimens based on the score are developed to optimize prognosis.

The role of radiotherapy in ovarian cancer remains uncertain, and current guidelines do not consistently recommend its use. Our study also showed no significant improvement in the prognosis of patients treated with radiotherapy compared to patients treated with chemotherapy alone. However, in instances involving lung metastases, we found no difference between the chemoradiotherapy alone and surgery groups. Radiotherapy alone may be considered in patients of this nature to mitigate surgical trauma and its associated complications. However, since this study was based on the SEER database and the number of patients of this type was only five, the conclusions obtained are somewhat limited. Also in cases with brain metastases, due to the small number of patients in the database, it was not possible to perform a valid statistical analysis. But some studies have shown that whole brain radiotherapy is the best option for patients with inoperable brain metastases [19]. And in patients with recurrent and refractory ovarian cancer, disease-free survival was prolonged in patients treated with radiotherapy [6]. Additionally, combining chemotherapy with whole abdominal radiotherapy (WART) holds promise for select ovarian cancer subtypes. Swenerton et al. published the results of a population-based study that compared six cycles of adjuvant standard-dose platinum-based chemotherapy with three cycles of chemotherapy followed by WART. They studied 703 patients with stage I-III ovarian cancer treated at British Columbia who had no significant residual disease after surgical staging. They found no difference in disease-specific survival between patients with plasma cancers treated with combination therapy or chemotherapy alone. However, in separate analyses of patients with stage I or II clear cell carcinoma, glioma-like tumours, or mucinous carcinoma, they found that disease-specific and overall survival was significantly better in patients who received combination therapy including WART than in patients who received chemotherapy alone [20]. Similarly, a 2012 study that included 241 patients with clear cell carcinoma of the ovary, the combination of WART and chemotherapy provided patients with a outcomes were significantly better than those who received chemotherapy alone, with an absolute improvement in 5-year disease-free survival of 20% (relative risk, 0.5) [7]. Consequently, further research into the use of radiotherapy in the management of ovarian cancer is warranted.

Our study shows that CA125-positive patients with stage IV epithelial ovarian cancer have a worse prognosis than CA125-negative patients. The reason for this may be that CA125 is associated with the progression of epithelial ovarian cancer. Research has indicated that elevated levels of CA125 hinder NK cell-mediated cytolysis and impede the destruction of ovarian cancer cells by NK cells [21–23]. Consequently, CA125-positive patients should be treated as early as possible. Our study suggests that CA125-positive patients are more likely to undergo surgery. Furthermore, post-treatment, monitoring the patient’s CA125 level serves as a valuable indicator of treatment response and facilitates the surveillance of residual lesions or the risk of recurrence [24]. Elevated postoperative CA125 concentrations exceeding 35 U/ml suggest residual lesions after tumour-reducing surgery, insensitivity to chemotherapeutic agents, and a heightened malignancy of the tumor [25]. In contrast, in the context of chemotherapy, maintaining CA125 levels below 35 U/ml, particularly after the first and third cycles of treatment, emerges as a pivotal determinant of prognosis among women with advanced ovarian cancer [26]. Lower serum CA125 concentrations and rapid normalization thereof signify a favorable response to chemotherapy, correlating with prolonged progression-free survival (PFS) [27]. Therefore, we recommend that CA125 levels should be monitored during diagnosis and treatment of patients with advanced ovarian cancer in order to choose the appropriate treatment modality.

We also found that the marital status of the patient affects the prognosis of ovarian cancer patients. Patients with a stable marital status exhibited improved prognosis and a reduced risk of mortality compared to their single, divorced, separated, or widowed counterparts. This result may be due to the fact that marital status is considered a major source of social support, and spousal support can positively influence the patient’s expectations about his/her own disease, leading to better coping with the diagnosis and treatment [28].The unmarried patients, including divorced/separated, widowed, and never-married, have a significantly increased risk of death after a diagnosis of ovarian cancer. And the widowed patients had the highest proportion of late diagnosis and the lowest proportion of surgical treatment. Marital status may be an independent factor in the death of patients with tumours in different studies. Furthermore, marital status may interact with tumor staging and treatment selection, exerting a substantial influence on overall prognosis [29].

SEER database provides longitudinal data on cancer patients, allowing us to track outcomes over time, such as survival rates, treatment patterns, and disease recurrence. Access to SEER data is often more cost-effective compared to conducting primary data collection, as it eliminates the need for expensive and time-consuming data collection efforts.While this database study provides a basis for the selection of treatment regimens for stage IV epithelial ovarian cancer patients, we must acknowledge its inherent limitations. Firstly, the sample size and representativeness of the database may be constrained, challenging the generalizability of the study findings. Secondly, biases or errors in the data collection process may have compromised the accuracy and credibility of the results. Additionally, due to constraints imposed by specific environments and conditions, the study findings may lack universality and require validation within a broader context. To address these limitations and ensure the reliability of the study results, multicenter data validation is warranted. By validating the findings across different regions, populations, and settings, we can ascertain the robustness of the study outcomes and gain a better understanding of their applicability and impact in real-world settings. This enhances the credibility and reliability of the research, providing a more solid foundation for further applications and decision-making processes.

## Conclusion

Our study showed that the prognosis of patients with stage IV epithelial ovarian cancer is affected by multiple factors, and the best prognosis is achieved with surgery plus adjuvant chemotherapy. In the presence of lung metastases that prevented surgery, a combination of radiotherapy and chemotherapy could be used. In the remaining cases, the addition of radiotherapy could not improve the prognosis of patients with stage IV epithelial ovarian cancer, and further studies are needed to determine the value of radiotherapy. This database study has certain limitations, and it needs to be verified by multi-center data in the real world.

## Data Availability

The datasets used and/or analysed during the current study are available from the corresponding author on reasonable request.

## References

[1] Siegel R, Giaquinto A, Jemal AJC. Cancer statistics, 2024. 2024, 74(1):12–49. 10.3322/caac.21820 38230766

[2] Robert LHJCL. Molecular characteristics and clinical behaviour of epithelial ovarian cancers. 2023, 555. 10.1016/j.canlet.2023.216057 36627048

[3] Michelle AR, Patricia EJAFP. Ovarian cancer: an overview. 2009, 80.

[4] Brian O, Robert PEJHOCNA. Diagnosis and Treatment of Ovarian Cancer. 2018, 32.

[5] González-Martín A, Harter P, Leary A, Lorusso D, Miller R, Pothuri B, Ray-Coquard I, Tan D, Bellet E, Oaknin A et al. Newly diagnosed and relapsed epithelial ovarian cancer: ESMO Clinical Practice Guideline for diagnosis, treatment and follow-up. 2023, 34(10):833–48. 10.1016/j.annonc.2023.07.011 37597580

[6] Nagai Y, Inamine M, Hirakawa M, Kamiyama K, Ogawa K, Toita T, Murayama S. Aoki YJGo: postoperative whole abdominal radiotherapy in clear cell adenocarcinoma of the ovary. 2007, 107(3):469–73. 10.1016/j.ygyno.2007.07.079 17765295

[7] Hoskins P, Le N, Gilks B, Tinker A, Santos J, Wong F, Swenerton KJJASCO. Low-stage ovarian clear cell carcinoma: population-based outcomes in British Columbia, Canada, with evidence for a survival benefit as a result of irradiation. 2012, 30(14):1656–62. 10.1200/JCO.2011.40.1646 22493415

[8] Orr B. Edwards RJHocoNA: diagnosis and treatment of Ovarian Cancer. 2018, 32(6):943–64. 10.1016/j.hoc.2018.07.010 30390767

[9] Di Donato V, Kontopantelis E, Aletti G, Casorelli A, Piacenti I, Bogani G, Lecce F. Benedetti Panici PJAoso: Trends in Mortality after primary cytoreductive surgery for ovarian Cancer: a systematic review and metaregression of Randomized clinical trials and observational studies. 2017, 24(6):1688–97. 10.1245/s10434-016-5680-7 27896508

[10] Deepa MN, Amanika K, Amy LW, Michaela EM, Carrie LL. William A CJGO: using an evidence-based triage algorithm to reduce 90-day mortality after primary debulking surgery for advanced epithelial ovarian cancer. 2019, 155. 10.1016/j.ygyno.2019.08.004 31402165

[11] Violante DD, Andrea G, Ottavia DO, Michele Carlo S, Anna DP, Margherita F, Francesca L, Giorgia P, Francesco B, Pasquale B et al. Hepatobiliary disease resection in patients with Advanced Epithelial Ovarian Cancer: Prognostic Role and Optimal Cytoreduction. 2020, 28.

[12] P AM, T DA BP, R S R FLALAP. S C, E C, S G : Randomised study of systematic lymphadenectomy in patients with epithelial ovarian cancer macroscopically confined to the pelvis. 2006, 95. 10.1038/sj.bjc.6603323 PMC2360519 16940979

[13] K PH, T G L GRH, A F-E AT. A dBJIJGC: pattern and clinical predictors of lymph node metastases in epithelial ovarian cancer. 2007, 17. 10.1111/j.1525-1438.2007.00931.x 17433064

[14] A F, A D, G G, A G, S G, A D, L F, E P, C S, F R et al: Secondary cytoreductive surgery for isolated lymph node recurrence of epithelial ovarian cancer: a multicenter study. 2014, 40. 10.1016/j.ejso.2013.11.026 24378007

[15] Pierluigi BP, Andrea G, Margherita F, Francesca L, Violante DDJCOR. Lymphadenectomy in Ovarian Cancer: Is It Still Justified? 2020, 22.

[16] Fairfield K, Lucas F, Earle C, Small L, Trimble E, Warren JJC. Regional variation in cancer-directed surgery and mortality among women with epithelial ovarian cancer in the Medicare population. 2010, 116(20):4840–8. 10.1002/cncr.25242 20578182

[17] Moore K, Reid M, Fong D, Myers T, Landrum L, Moxley K, Walker J, McMeekin D. Mannel RJGo: ovarian cancer in the octogenarian: does the paradigm of aggressive cytoreductive surgery and chemotherapy still apply? 2008, 110(2):133–9. 10.1016/j.ygyno.2008.03.008 18495221

[18] Falandry C, Weber B, Savoye A, Tinquaut F, Tredan O, Sevin E, Stefani L, Savinelli F, Atlassi M, Salvat J et al. Development of a geriatric vulnerability score in elderly patients with advanced ovarian cancer treated with first-line carboplatin: a GINECO prospective trial. 2013, 24(11):2808–13. 10.1093/annonc/mdt360 24061628

[19] Eifel, PJBp. Obstetrics rC, gynaecology: role of radiation therapy. 2017, 41:118–25. 10.1016/j.bpobgyn.2016.11.005 27986398

[20] Swenerton K, Santos J, Gilks C, Köbel M, Hoskins P, Wong F. Le NJAooojotESfMO: Histotype predicts the curative potential of radiotherapy: the example of ovarian cancers. 2011, 22(2):341–7. 10.1093/annonc/mdq383 PMC3030466 20693298

[21] Patankar M, Jing Y, Morrison J, Belisle J, Lattanzio F, Deng Y, Wong N, Morris H, Dell A. Clark GJGo: potent suppression of natural killer cell response mediated by the ovarian tumor marker CA125. 2005, 99(3):704–13. 10.1016/j.ygyno.2005.07.030 16126266

[22] Felder M, Kapur A, Rakhmilevich A, Qu X, Sondel P, Gillies S, Connor J. Patankar MJGo: MUC16 suppresses human and murine innate immune responses. 2019, 152(3):618–28. 10.1016/j.ygyno.2018.12.023 PMC8327469 30626487

[23] Gubbels J, Felder M, Horibata S, Belisle J, Kapur A, Holden H, Petrie S, Migneault M, Rancourt C, Connor J et al. MUC16 provides immune protection by inhibiting synapse formation between NK and ovarian tumor cells. 2010, 9:11. 10.1186/1476-4598-9-11 PMC2818693 20089172

[24] Zhang M, Cheng S, Jin Y, Zhao Y, Wang YJBR. Roles of CA125 in diagnosis, prediction, and oncogenesis of ovarian cancer. 2021, 1875(2):188503. 10.1016/j.bbcan.2021.188503 33421585

[25] Bottoni P. Scatena RJAiem, biology: the role of CA 125 as tumor marker: biochemical and clinical aspects. 2015, 867:229–44. 10.1007/978-94-017-7215-0_14 26530369

[26] Lee M, Chang M, Yoo H, Lee K, Chay D, Cho H, Kim S, Kim Y. Kim JJYmj: clinical significance of CA125 level after the First cycle of Chemotherapy on Survival of patients with Advanced Ovarian Cancer. 2016, 57(3):580–7. 10.3349/ymj.2016.57.3.580 PMC4800345 26996555

[27] Zhang D, Jiang Y, Luo S, Zhou R, Jiang Q. Linghu HJCca, chemistry ijoc: serum CA125 levels predict outcome of interval debulking surgery after neoadjuvant chemotherapy in patients with advanced ovarian cancer. 2018, 484:32–5. 10.1016/j.cca.2018.04.030 29702068

[28] Kanters A, Morris A, Abrahamse P, Mody L. Suwanabol PJDotc, rectum: The Effect of Peer Support on Colorectal Cancer Patients’ Adherence to Guideline-Concordant Multidisciplinary Care. 2018, 61(7):817–23. 10.1097/DCR.0000000000001067 PMC5992023 29771795

[29] Luo P, Zhou J, Jin S, Qing M, Ma HJJ. Influence of marital status on overall survival in patients with ovarian serous carcinoma: finding from the surveillance epidemiology and end results (SEER) database. 2019, 12(1):126. 10.1186/s13048-019-0600-7 PMC6937688 31888704

